# When Should Oncologists Use the Words *Hope* and *Cure*?

**DOI:** 10.1093/jncics/pkaa066

**Published:** 2020-08-14

**Authors:** Belinda E Kiely, Martin R Stockler

**Affiliations:** NHMRC Clinical Trials Centre, The University of Sydney, Camperdown, NSW, Australia

Advances in cancer diagnosis, screening, and treatment have substantially improved the 5-year survival rates of most cancers over the last 30 years. Long-term survivors of cancer are increasingly common, and research is increasingly directed at helping people live well after a diagnosis of cancer. These advances have provoked optimism among oncologists, but many still hesitate to use the word *cure* with their patients.

In their article published in this issue of the Journal, Corn and colleagues (1) tested the hypothesis that oncologists are reluctant to use the terms *hope* and *cure* in journal articles. The authors analyzed 13 363 articles classified as primary research, editorials, or narrative essays published in the *Journal of Clinical Oncology* (from 2000 to 2018) or *JAMA Oncology* (from 2015 to 2019) and counted the numbers and proportions of articles, and of sentences, that included the words *hope* or *cure*. Overall, the words *hope* and *cure* were used infrequently and mentioned less often in primary research articles than narrative essays. Use of both terms diminished over time despite increasing cancer survival rates. The investigators concluded that the time has come for oncologists to embrace the words *hope* and *cure*.

To consider this, it is necessary to distinguish how oncologists communicate in journal articles vs how they communicate to patients. Journal articles typically describe groups of people, and the terminology and syntax are aimed at a target audience of other oncologists. Given neither *hope* nor *cure* has an accepted definition in oncology, it is not surprising these words are seldom used in publications. When oncologists communicate with patients, the audience is an individual with cancer and the language used needs to be clear, easy to understand, and reassuring.

In cancer care, *hope* has been defined as “the confident but uncertain expectation of a future good that appears realistically possible and is personally significant to the individual” (2). Having things to hope for is important for all people affected by cancer, and *cure*, or at least prolonged survival, is often the primary target of that hope. Oncologists are able to foster and deplete *hope* through their communication of information about diagnosis, treatment, and prognosis. In the setting of advanced cancer, oncologists can help patients redirect their *hope*, for example, towards hoping for dignity, symptom relief, and time with their family. It is unlikely that use of the word *hope* is necessary to convey hope. For example, physician behaviors identified by patients as hope-giving include combining honesty and empathy, framing hope in a wider context than *cure*, and emphasizing achievable treatment goals (3).

Knowing when and how to use the words *cure* and *cured* in conversations with people affected by cancer is much more complicated. The American Society of Clinical Oncology defines *cancer cure* for an individual in its cancer survivorship booklet as “when a person’s cancer has not returned for at least 5 years after treatment” (4). Another definition of *cancer cure* is when the mortality rate of people diagnosed with a certain cancer returns to the level expected in the general population of the same sex and age (5). For most cancers, this is much longer than 5 years after treatment. For example, women with estrogen receptor–positive, early-stage breast cancer have a persistent risk of recurrence and death from breast cancer for at least 20 years after their initial diagnosis (6). Before encouraging oncologists to use the word *cure* more often, we need a better understanding of what *cure* means to oncologists and patients. For example, should there be an agreed threshold for the time from diagnosis and/or estimated risk of recurrence beyond which oncologists should be comfortable using the word *cure*?

In a survey of 117 American oncology clinicians, 81% responded that they were “hesitant to tell a patient that they are cured,” and 63% responded that they “would never tell a patient that they are cured” (7). Only 36% of respondents were “comfortable telling a patient that they are cured within 0-5 years after completing the initial phase of their treatment,” with most respondents wanting 6-10 years or longer before using the word *cured* (7).

Conversations between oncologists and their patients with an early-stage cancer stereotypically focus on the risk of recurrence with and without additional treatment. Many of these patients are cured, but their risk of future recurrence—even when very small—makes oncologists reluctant to use the word *cure*, fearing it will be interpreted as a promise, and particularly one that might be broken. Instead, we have become familiar using less specific words and phrases, including *curable*, *curative intent*, *in remission*, *no evidence of disease*, *long-term survivor*, and *most likely cured*.

Avoiding the word *cured* may cause some patients uncertainty, fear about the future, and excessive worry. A recent systematic review identified "help coping with the fear of cancer recurrence" as the most prevalent and severe unmet need reported by Australian cancer patients (8). Using the word *cured* in selected patients may increase their confidence about the future and might help them cope better with the physical and psychosocial aftermath of their diagnosis and treatment.

The benefit of using the word *cured* with people affected by an early-stage cancer is to provide hope and reassurance, but the harm is misleading the minority who will suffer a recurrence. On the other hand, never using the word *cured* instills unnecessary fear and uncertainty in all. Cancer recurrence will always be traumatic, and it is difficult to determine whether this trauma is worsened if an oncologist previously claimed the cancer was cured.

Oncologists may be more comfortable using the word *incurable* in advanced cancer. A survey of 206 medical oncologists in Australia and New Zealand found that 88% reported explaining that “the cancer is incurable” to all their patients with incurable cancer (9). Despite this, many people with advanced cancer misunderstand their prognosis and the aims of their treatment. For example, a survey of Americans cancer patients found that only 31% of those with advanced lung cancer and 19% of those with advanced colorectal cancer reported that chemotherapy was not at all likely to cure their cancer (10). Even when patients understand their situation and accept that they are dying, they often continue to hope for a cure. Clayton et al. (11) found that Australian palliative care patients may simultaneously hope for a cure and acknowledge the terminal nature of their illness. Perhaps hoping for prolonged survival or the unlikely possibility of cure is acceptable if accompanied by a simultaneous and accurate understanding of prognosis and suitable preparations for deterioration and death.

Both *hope* and *cure* have different meanings for individual patients, oncologists, and contexts. *Hope* is essential for all human beings, including patients and their oncologists. Oncologists should communicate in a way that fosters and sustains realistic hope even in the setting of advanced cancer. Although the words *cure* and *cured* should not be used at all with many people affected by cancer, there must be some in whom the type, stage, course, and disease-free interval warrant use of the word *cured*. More thought, research, discussion, and consensus are required to help guide oncologists about when it is reasonable and appropriate to use the word *cured*.

## Notes

**Disclosures:** The authors have no conflicts of interest to disclose relevant to this article. BEK has received conference support, speaker’s honorariums, and advisory board honorariums from Roche; and advisory board honorariums from Novartis and Teva.

**Author contributions:** Both authors drafted, revised and approved the final version.

## Data availability

Not applicable for this manuscript as no data involved.
